# Schema Enforcement and Structured-Output Stability in Locally Deployed LLMs for Clinical Admission-Note Editing: A Proxy-Based Pre-Deployment Evaluation

**DOI:** 10.3390/healthcare14142150

**Published:** 2026-07-16

**Authors:** Ya-Lun Yang, Chia-Jung Chen, Tin-Kwang Lin, Shih-Chun Lin, Malcolm Koo

**Affiliations:** 1 Department of Nursing, Dalin Tzu Chi Hospital, Buddhist Tzu Chi Medical Foundation, Dalin 622401, Chiayi, Taiwan; 2 Division of Cardiology, Department of Internal Medicine, Dalin Tzu Chi Hospital, Buddhist Tzu Chi Medical Foundation, Dalin 622401, Chiayi, Taiwan; 3 School of Medicine, Tzu Chi University, Hualien City 970374, Hualien, Taiwan; 4 School of Nursing, National Taipei University of Nursing and Health Sciences, Taipei City 112303, Taiwan; 5 Department of Medical Research, Dalin Tzu Chi Hospital, Buddhist Tzu Chi Medical Foundation, Dalin 622401, Chiayi, Taiwan; 6 Dalla Lana School of Public Health, University of Toronto, Toronto, ON M5T 3M7, Canada

**Keywords:** large language models, clinical documentation, JSON validity, local deployment, nursing informatics, generative artificial intelligence

## Abstract

**Background:** Reliable structured-output generation is a prerequisite for using large language models (LLMs) in automated clinical documentation workflows, but many evaluations focus on clinical quality before testing whether outputs are parseable, schema-compliant, and stable. **Methods:** We evaluated three locally deployed open-weight LLMs in the 7- to 8-billion-parameter range (Llama3-Med42-8B, Meta-Llama-3-8B-Instruct, and Mistral-7B-Instruct-v0.3) for structured admission-note editing. Seventy de-identified English-language admission notes (35 internal medicine and 35 surgical) were processed by each model in three independent runs under two output-control conditions: a free-text JSON prompt and a schema-enforced structured-output condition. A total of 1260 local inferences were performed in LM Studio on consumer-grade hardware. Automated proxy metrics assessed JSON/schema validity, run-to-run stability, instruction compliance, verbosity, numeric-token preservation, and uncertainty-marker change without clinician adjudication of clinical correctness. **Results:** Under the free-text JSON prompt, the tested Mistral-7B-Instruct-v0.3/embedded-prompt configuration had the weakest structural reliability (74.3–78.6% first-pass validity per run; 18.6–21.4% persistent parse/schema failures after retry), with at least one final failure for 17 of 70 notes. In a message-format sensitivity analysis using Meta-Llama-3-8B-Instruct, embedding system instructions in the user message increased first-attempt invalid outputs compared with separate system/user roles (55/700, 7.9% vs. 12/700, 1.7%). Under schema enforcement, all models produced 70 of 70 first-pass valid, schema-compliant outputs in every run. Documentation behavior nevertheless differed by model, including differences in verbosity and numeric-token preservation. **Conclusions:** Schema enforcement removed parsing failures in this sample but did not eliminate model-specific editing behavior. Proxy-based screening can identify structurally unstable model-prompt or model-format configurations before clinician review.

## 1. Introduction

Large language models (LLMs) are increasingly explored for clinical documentation tasks, including the generation, revision, and restructuring of medical records [1]. Commercial tools such as Google’s Med-PaLM 2 [2] and Microsoft’s Dragon Copilot [3] have been developed for clinical-note writing, and a growing body of research supports the potential of generative artificial intelligence (AI) to reduce documentation burden while maintaining or improving record quality [4,5,6]. Systematic reviews have reported that AI-assisted documentation can decrease the time clinicians spend on record-keeping [4], improve the grammatical and structural quality of notes [5], and enhance readability across clinical settings [6]. In Taiwan, as in many healthcare systems, nurse practitioners bear substantial responsibility for writing admission notes under time pressure, making AI-assisted documentation a practical area for evaluation.

Most evaluations of LLMs for clinical documentation focus on output quality, such as whether the generated or revised text is accurate, complete, readable, and clinically appropriate [6,7]. These assessments are essential but usually require expert clinician review and are resource-intensive by design. Before such review can be performed efficiently, however, a more basic deployment-level question must be addressed: whether the model can produce structurally valid, stable, and behaviorally predictable outputs under the intended deployment conditions. A model that intermittently fails to generate parseable structured outputs or that produces different output statuses across repeated runs on identical inputs, introduces operational risks that are distinct from clinical content quality. In automated documentation pipelines, malformed or schema-noncompliant outputs can interrupt downstream processing, require manual intervention, or necessitate retry and fallback logic.

### Related Work and Study Rationale

Recent work on clinical LLMs can be grouped into four related areas: clinical documentation and summarization, medical LLM benchmarks and healthcare LLM systems, local or privacy-preserving deployment, and structured-output generation. Table 1 summarizes these areas and situates the present study relative to prior work.

First, studies of AI-assisted clinical documentation and summarization have mainly emphasized semantic or clinical endpoints, including factual accuracy, completeness, readability, extraction performance, summarization quality, or clinician-rated documentation quality [4,5,6,7]. These outcomes are essential for assessing whether LLM-generated text is clinically acceptable. However, they do not directly determine whether model outputs are structurally valid and pipeline-ready for automated documentation workflows.

Second, benchmark-based evaluations and healthcare LLM system studies provide useful evidence about medical knowledge, reasoning, question-answering, and task performance [1,2,3]. These studies help characterize the general medical capabilities of LLMs, but benchmark performance may not translate directly into reliable behavior in a local documentation pipeline. In particular, high benchmark performance does not necessarily ensure JavaScript Object Notation (JSON) validity, schema adherence, instruction compliance, or run-to-run stability when models are used for structured clinical-note editing.

Third, local and privacy-preserving LLM deployment has become increasingly relevant in healthcare because it can reduce the need to transmit patient data to external servers [8,9]. However, local deployment on institutional or consumer-grade hardware introduces constraints that differ from cloud-based application programming interface (API) access, including limited computational resources, quantized model weights, model-specific chat-template requirements, and limited built-in structured-output controls. These constraints are particularly relevant for hospitals or research teams using consumer-oriented local inference tools, such as LM Studio (Element Labs Inc., New York, NY, USA) or Ollama (Ollama, Inc., San Francisco, CA, USA), rather than enterprise-level cloud systems with built-in structured-output controls.

Fourth, structured-output generation has emerged as a practical reliability issue for LLM-based applications. Recent work on structured clinical-note extraction has shown that small locally deployable language models may produce malformed structured responses and that parseability can vary according to output format, prompt specificity, document length, note type, and model size [10]. More broadly, format-restricted generation studies have shown that requiring models to follow strict formats such as JSON, Extensible Markup Language (XML), or YAML (originally “Yet Another Markup Language” and now commonly expanded as “YAML Ain’t Markup Language”) can alter task performance, indicating that structured-output generation should not be treated as a neutral implementation detail [11,12]. Constraint-decoding and grammar-guided generation methods can improve schema adherence by enforcing output structure during generation [13,14], but these methods are not universally available in consumer-oriented local deployment workflows and may require separate evaluation of both structural validity and output quality.

Therefore, clinical quality and structured-output reliability should be treated as related but separate dimensions of model evaluation. Structured-output reliability should be evaluated as a distinct pre-deployment requirement before models proceed to clinician-led assessment of clinical correctness, safety, and usability.

This study aimed to address the gap between clinical-quality evaluation and deployment-readiness testing by evaluating the pre-deployment suitability of three locally deployed LLMs in the 7- to 8-billion-parameter range for structured admission-note editing using automated proxy metrics. Rather than assessing clinical correctness or documentation quality, which require expert human review, we focused on whether locally deployed open-weight LLMs can produce structurally valid, schema-compliant, and stable outputs under a practical admission-note editing workflow. Specifically, we examined two dimensions of operational behavior: structured-output stability, defined as the ability to consistently produce complete and parseable JSON outputs across repeated runs under identical conditions, and documentation behavior, defined as the model’s characteristic patterns of verbosity, instruction compliance, and content modification when rewriting clinical notes. We also compared a free-text JSON prompt with a schema-enforced structured-output condition to determine whether local response-format control could reduce parsing failures without eliminating documentation-behavior differences.

This study makes three contributions. First, it proposes and applies a proxy-based pre-deployment screening framework, an evaluation methodology rather than a new model or decoding algorithm, which separates structured-output stability from downstream clinical content quality. Second, it uses repeated inference runs on identical clinical notes to detect stochastic output instability and to assess whether single-run evaluation is sufficient to characterize deployment behavior. Third, it compares three locally feasible open-weight models under the same admission-note editing task, including two Llama 3-based models with the same base architecture but different fine-tuning strategies and one cross-architecture comparator. By evaluating 70 admission notes across three independent runs for each model, we sought to determine whether automated proxy-based screening can identify structurally unstable models and reveal deployment-relevant differences in parseability, instruction compliance, verbosity, and content-modification signals before resource-intensive clinician evaluation. Given the model-specific chat-template constraint observed for Mistral-7B-Instruct-v0.3, we also conducted a targeted message-format sensitivity analysis using Meta-Llama-3-8B-Instruct to examine whether embedding system instructions within the user message could affect structured-output reliability when model identity was held constant.

The remainder of this paper is organized as follows. Section 2 describes the study design, clinical-note evaluation set, model-selection rationale, local inference environment, prompt structure, output-cleaning procedure, and proxy-based evaluation metrics. Section 3 presents the structured-output stability results, documentation-behavior profiles, and model-level screening interpretation. Section 4 discusses the implications of these findings for local clinical LLM deployment, the role of proxy-based screening before clinician review, and the limitations of this study. Section 5 summarizes the main conclusions.

## 2. Materials and Methods

### 2.1. Study Design

This study employed a repeated-measures observational design to evaluate the pre-deployment suitability of three locally deployed LLMs for structured admission-note editing. Each model processed the same set of 70 admission notes across three independent runs under two output-control conditions: a free-text JSON prompt and a schema-enforced structured-output condition. Decoding parameters were held constant across models, runs, and output-control conditions. Model behavior was assessed using automated proxy metrics without human evaluation of clinical content. This study was designed as a pre-deployment screening assessment, focusing on operational reliability and behavioral characterization rather than clinical quality or safety.

**Figure 1:**
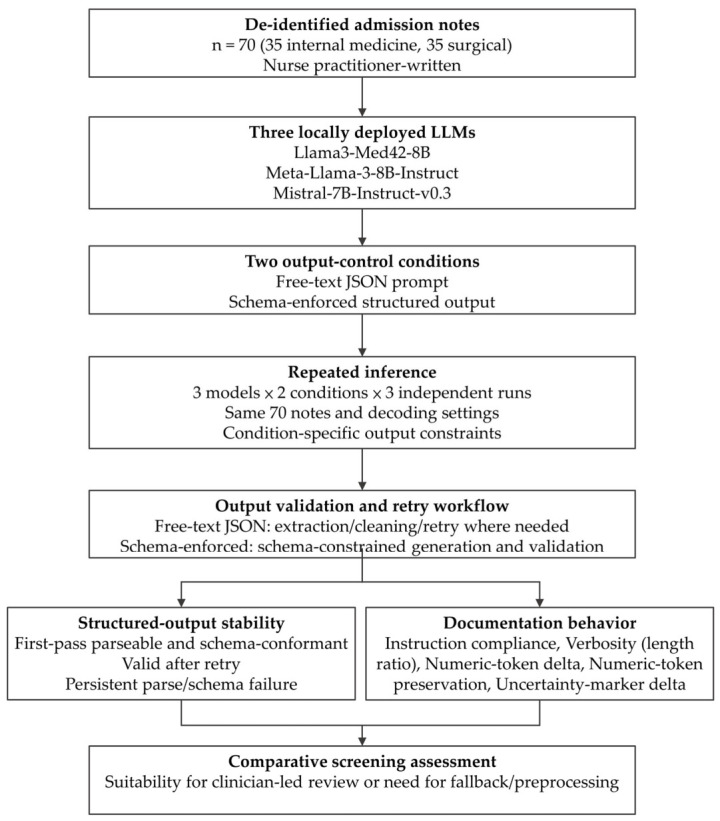
Pre-deployment screening workflow for the clinical-note evaluation set under free-text JSON and schema-enforced structured-output conditions.

The retrospective computational workflow consisted of five steps: selection of eligible admission notes, de-identification, conversion to plain text, local model inference under the two output-control conditions, and automated analysis of model outputs using scripted proxy metrics. The model outputs were not used for patient care, and no clinical decisions were made from the generated text. All processing was performed locally, and no clinical documents or model outputs were transmitted to external model providers. The overall pre-deployment screening workflow is summarized in Figure 1.

### 2.2. Clinical Notes

Seventy de-identified English-language hospital admission notes were retrospectively selected from routine clinical documentation at a regional teaching hospital in southern Taiwan. All notes were originally written by nurse practitioners as part of usual clinical care before de-identification. Eligible notes were admission notes from either internal medicine or surgical services that contained sufficient clinical content for editing, including the reason for admission, relevant history, examination findings, and an initial diagnostic or management plan. Notes were excluded if they were duplicate records, incomplete notes, non-admission notes, non-English notes, or notes that still contained identifiable information after de-identification.

The evaluation set was stratified by clinical service and included 35 internal medicine notes and 35 surgical notes. This balanced allocation was chosen to include two common admission-note contexts with different documentation patterns. Internal medicine notes often include multiple comorbidities, medication histories, laboratory values, and diagnostic uncertainty, whereas surgical notes more commonly emphasize procedural indications, perioperative planning, and anatomical or operative details. The 70 notes were used only as an evaluation set for pre-deployment screening; no model training, fine-tuning, or parameter updating was performed.

Before analysis, the source documents were checked to ensure that patient names, identification numbers, admission dates, and other personally identifiable information were not present. The de-identified notes were converted to plain-text files and used as model inputs without editing of clinical content. The dataset was designed to assess model behavior before deployment within this local documentation context.

To characterize the evaluation set, source-note length, numeric-token count, and uncertainty-marker count were summarized before model inference. These descriptive characteristics were used to describe the input notes and to support interpretation of later proxy metrics related to verbosity, quantitative-content change, and diagnostic-certainty language. The operational definitions of these metrics are provided in Section 2.5.

### 2.3. Models and Inference Environment

Three open-weight LLMs in the 7- to 8-billion-parameter range were selected for evaluation (Table 2). Model selection was guided by practical deployment feasibility and by the study objective of comparing structured-output reliability and documentation behavior under local inference conditions. First, the 7–8 billion parameters were selected because models of this size can be loaded and executed on consumer-grade graphics processing units (GPUs) with 16 GB video random access memory (VRAM) after 4 bit quantization, making them relevant to institutions without dedicated server infrastructure. This configuration reflects a practical local deployment scenario for hospitals or research teams that require local data processing but do not have access to high-performance server clusters or cloud-based inference services.

Second, two models sharing the same base architecture (Llama 3 8B) but differing in fine-tuning strategy were included to enable a controlled comparison: Llama3-Med42-8B [15], fine-tuned on medical-domain data by M42 Health, and Meta-Llama-3-8B-Instruct [16], fine-tuned for general-purpose instruction following by Meta. This pairing allowed comparison of domain-specific and general instruction-tuned behavior while holding the base architecture constant.

Third, Mistral-7B-Instruct-v0.3 [17] was included as a cross-architecture comparator at a similar parameter scale, allowing assessment of whether the observed deployment behavior was specific to Llama 3-based models.

Fourth, all three models are publicly available in GPT-Generated Unified Format (GGUF) from Hugging Face repositories, can be run using consumer-oriented inference tools such as LM Studio without specialized infrastructure, and are therefore representative of models that resource-constrained institutions could plausibly deploy.

The selection prioritized ecological validity over comprehensiveness; the aim was to construct a minimal comparison that demonstrates the framework’s capacity to differentiate models on deployment-relevant dimensions, rather than to survey all available models. For local inference, 4 bit Q4_K_S quantized variants were used to fit the available GPU memory. No fine-tuning, model training, or parameter updating was performed in this study. The models were evaluated as publicly available open-weight models, and the fine-tuning domains reported in Table 2 refer to the original model-development process described by the model providers rather than any training performed by the present research team.

All experiments were conducted on a consumer-grade desktop system MAG Infinite S3 desktop computer (Micro-Star International Co., Ltd., New Taipei City, Taiwan) equipped with an Intel Core i7-14700F processor (2.10 GHz base clock), 64 GB double data rate random access memory (DDR RAM), and an NVIDIA GeForce RTX 5060 Ti GPU with 16 GB GDDR7 VRAM, running Microsoft Windows 11 version 24H2. Models were loaded and executed using LM Studio version 0.4.1, accessed programmatically via its OpenAI-compatible local API endpoint on port 1234 (http://localhost:1234/v1). This local API workflow allowed standardized programmatic inference while retaining all model execution and clinical-note processing within the local computer environment.

### 2.4. Prompt Design and Inference Parameters

The prompt was developed through author consensus to reflect the intended clinical documentation task and was subsequently refined and shortened with AI assistance to improve clarity and reduce unnecessary wording. The final prompt was designed to satisfy two requirements: clinical-note revision and structured-output reproducibility.

Each admission note was submitted using a structured prompt with four components: (1) a task instruction asking the model to edit and improve the wording and structure of nurse practitioner-written admission notes without adding, deleting, or changing clinical facts; (2) an instruction to preserve diagnostic certainty and avoid adding new treatment recommendations; (3) a required four-field JSON schema; and (4) the source admission note. The required JSON object contained four fields: revised_note, defined as the edited admission note in string format; change_summary, defined as an array of strings describing the main wording or structural changes; missing_or_unclear_information, defined as an array of strings identifying information gaps or ambiguities in the source note; and safety_flags, defined as an array of strings identifying potential clinical safety concerns. The prompt explicitly instructed the model to return only one valid JSON object, without code fences, preamble text, explanations, or commentary outside the JSON structure.

The same informational prompt content was used for all three models and in both output-control conditions. In the free-text JSON condition, the JSON schema was provided only in the prompt, and the model response was parsed after generation. In the schema-enforced structured-output condition, the same four-field schema was also supplied through the local API response-format mechanism available in LM Studio. This mechanism was configured as a strict JSON schema (strict = true, additionalProperties = false) specifying the four required fields and their expected data types.

The prompt content was packaged according to each model’s local chat-template requirements. For Llama3-Med42-8B and Meta-Llama-3-8B-Instruct, the task instructions and JSON schema requirements were placed in the system message, whereas the source admission note was placed in the user message, which also restated the four required keys and their expected types. In this context, “accepted” means that the LM Studio OpenAI-compatible local API call completed using separate system and user roles without a chat-template error. For Mistral-7B-Instruct-v0.3, the same task instructions, schema requirements, and source note were combined into a single user message because the local chat template did not support a separate system role. The system-equivalent instructions were placed at the beginning of the user message under a bracketed header before the source note. This model-specific adjustment was necessary to avoid local API errors and was treated as a practical deployment constraint when using open-weight models with different chat-template requirements. The prompt was not separately optimized for each model; instead, a common institutional prompt with equivalent informational content was used to support comparability across models. The exact prompt templates, output-cleaning rules, schema-enforcement configuration, and annotated R code are provided in Supplementary File S1.

Inference parameters were held constant across all models, runs, and output-control conditions: temperature = 0.2, maximum generated-output tokens = 1024, and streaming disabled. Temperature was set to a low but nonzero value to reduce sampling variability while retaining the possibility of stochastic variation that the repeated-run design was intended to detect. The 1024-token limit applied to generated output rather than to the input context. It was treated as a potential deployment constraint and was checked during output validation to determine whether structured responses appeared to be truncated by the cap.

Under these settings, each model processed all 70 notes in a single sequential pass under each output-control condition, and this procedure was repeated three times independently for each model. This yielded 1260 model inferences in total: 3 models × 70 notes × 3 runs × 2 output-control conditions.

The three repeated runs were selected as a pragmatic repeated-execution design to evaluate run-to-run stability under identical deployment conditions. Because inference was performed at a nonzero temperature, stochastic variation could occur even when the input note, prompt, model, output-control condition, and inference parameters were unchanged. A single-run evaluation would identify only first-pass structured-output validity and could miss instability that appears only across repeated executions. By repeating the full 70-note evaluation three times for each model and condition, model behavior could be classified at the aggregate and per-note levels, distinguishing outputs that were valid in all three runs, failures observed in all three runs, and statuses that varied across runs. This design was intended as a practical pre-deployment stability check rather than a formal power-based estimate of rare failure rates.

#### Message-Format Sensitivity Analysis Methods

Because Mistral-7B-Instruct-v0.3 required the system-equivalent instructions to be embedded in the user message, model identity and message format were not fully separable in the primary free-text JSON comparison. To assess whether the embedded-user format itself could affect structured-output reliability, we conducted a targeted message-format sensitivity analysis using Meta-Llama-3-8B-Instruct. This model was selected because it accepted separate system and user roles in the primary analysis and provided a general instruction-tuned comparator at a similar parameter scale.

The same free-text JSON prompt was tested under two message configurations: separate system and user roles versus system instructions embedded at the beginning of the user message. The informational prompt content, admission notes, temperature, maximum output length, retry rule, JSON extraction procedure, and schema-validation rules were otherwise unchanged. Each of the 70 admission notes was processed 10 times under each message format, yielding 1400 primary sensitivity-analysis outputs before retry. The order of message formats was randomized within note-run pairs. This analysis was designed to isolate the effect of message format within one model; it did not fully separate message-format effects from possible model-by-format interactions involving Mistral.

### 2.5. Proxy-Based Evaluation Metrics

Model outputs were evaluated using automated proxy metrics across two deployment-relevant domains: structured-output stability and documentation behavior. These metrics were selected to support scalable and reproducible pre-deployment screening. Structured-output stability assessed whether model responses could be parsed into the required JSON schema across repeated runs and output-control conditions. Documentation behavior assessed changes in output length, numeric content, and diagnostic uncertainty language among structurally valid outputs. All metrics were computed automatically in R from the stored raw model responses and the source notes, with no human annotation or manual editing of model output. The exact extraction rules and regular expressions are summarized in Supplementary Table S1 and implemented in the annotated code in Supplementary File S1. These metrics were used to identify operational fragility and content-modification signals, without assessing clinical correctness, safety, or usability through human review.

#### 2.5.1. Structured-Output Stability

For each inference, the raw model response was stored before any cleaning or parsing. The same automated parsing workflow was applied to both output-control conditions. First, the first balanced JSON object in the response was extracted by scanning from the first opening brace to its matching closing brace, with brace counting that ignored braces inside quoted strings; any leading or trailing text outside this object, including Markdown code fences, was thereby discarded. Second, common formatting artifacts that prevented parsing, such as triple-quoted string values in the revised_note field, were repaired using predefined string-processing rules. Third, the extracted object was parsed using jsonlite in R. Fourth, the parsed object was checked for schema conformance: it had to contain exactly the four required keys, revised_note, change_summary, missing_or_unclear_information, and safety_flags, with no key missing and no additional key present; revised_note had to be a single string; and change_summary, missing_or_unclear_information, and safety_flags each had to be an array whose elements were all strings. For the schema-enforced condition, this workflow served as an independent verification step after generation rather than a replacement for response-format control.

Outputs were classified as ok when the raw or cleaned first-pass response was parseable and schema-compliant, ok_after_retry when a second inference attempt produced a parseable and schema-compliant response after initial failure, and fail when the output remained unparseable or schema-noncompliant after retry. No manual editing of clinical content was performed during output cleaning.

Structured-output stability was summarized at both the run and note levels within each output-control condition. At the run level, output-status distributions were compared across the three independent runs for each model. At the note level, the status assigned to each admission note was compared across runs. Notes were classified as consistently valid if they produced valid structured output in all three runs, consistently failed if they resulted in persistent failure in all three runs, and variable if their status changed across runs. This note-level classification was used to detect stochastic instability that could be missed by aggregate validity rates alone.

#### 2.5.2. Output and Documentation Behavior

Among outputs classified as structurally valid, documentation behavior was characterized using five automated proxy metrics: instruction compliance, verbosity profile, numeric-token delta, numeric-token preservation, and uncertainty-marker delta. A schema-validity content check was also applied.

Instruction compliance was assessed by recording whether the raw output included code-fence markers, such as ```json, or leading text before the JSON object. These elements violated the prompt instruction to return only a JSON object and were treated as signals of partial instruction noncompliance. Although these outputs could sometimes be recovered through cleaning, their presence indicated increased fragility in automated pipelines.

Verbosity profile was quantified as the character-level length ratio between the revised note and the original input note. For each valid output, the number of characters in the revised_note field was divided by the number of characters in the corresponding source admission note. Character counts were calculated after removing whitespace so that differences in line breaks, indentation, or spacing introduced by the model would not influence the metric. A ratio below 1.0 indicated compression relative to the source note, a ratio close to 1.0 indicated a length-neutral revision, and a ratio above 1.0 indicated expansion. The median length ratio and distribution across notes were computed for each model.

Numeric-token delta was calculated as the difference between the number of numeric tokens in the revised note and the number of numeric tokens in the corresponding original note. Numeric tokens were extracted automatically with the regular expression \b\d+(\.\d+)?\b, which matches integer or decimal digit sequences delimited by word boundaries. Because a digit fused to adjacent letters has no neighboring word boundary, digits embedded in alphanumeric clinical terms or staging and mutation codes, such as T2DM or HbA1c, were not matched. When a non-word character separated digits from letters, the digits were matched; for example, the 19 in COVID-19, and each numeric component of a hyphenated date or range, were counted as separate numeric tokens, and a value joined to its unit was counted only when a non-word character separated the number from the unit. This extraction was applied identically to the source and revised notes. A negative delta indicated a reduction in numeric tokens, a delta of zero indicated no change, and a positive delta indicated an increase in numeric tokens. This metric was interpreted as a proxy signal of possible quantitative-content modification rather than as direct evidence of clinical error.

Numeric-token preservation was calculated as the percentage of source numeric tokens that reappeared in the revised note. It was computed as the multiset intersection of the source and revised numeric-token sets, divided by the number of source numeric tokens, multiplied by 100. Matching was based on token value with multiplicity and was independent of position within the note. A higher value indicated that more of the original numeric content was retained. This metric was interpreted as a proxy signal of quantitative-content retention rather than as a measure of clinical accuracy.

Uncertainty-marker delta was calculated as the difference between the number of uncertainty markers in the revised note and the number in the corresponding original note. Uncertainty markers were counted using a predefined case-insensitive regular expression matching the whole-word terms “possible,” “possibly,” “suspect,” “suspected,” “likely,” “consider,” “r/o,” and “rule out,” with one additional count added for each question mark in the text. The count for a note was the number of regular-expression matches plus the number of question marks. A negative delta indicated reduced uncertainty language, whereas a positive delta indicated increased uncertainty language. This metric was interpreted as a proxy signal of possible change in expressed diagnostic certainty rather than as a direct measure of clinical appropriateness.

In addition, each schema-valid output was checked for low-content responses. An output was flagged as schema-valid but low-content if the revised_note field was empty or contained only a placeholder token (for example, “NA,” “none,” “not applicable,” or “unknown”), or if the array fields were empty or contained only placeholder strings. This check identified outputs that passed schema validation while carrying no usable revised text, and was reported separately from the other proxy metrics.

### 2.6. Data Analysis

All metrics were computed programmatically using R version 4.5.2 with the packages fs, httr2, jsonlite, stringr, readr, dplyr, tidyr, tibble, and purrr. Proxy metrics were summarized at the model, condition, run, and note levels. Categorical outcomes, including output-status categories, instruction-compliance indicators, and note-level stability classifications, were reported as counts and proportions. Continuous or ordinal metrics, including length ratio, numeric-token delta, numeric-token preservation, and uncertainty-marker delta, were summarized using medians, interquartile ranges, and ranges where appropriate. Cross-run stability was described using run-level outcome distributions and note-level consistency classifications.

Exploratory paired comparisons between the free-text JSON and schema-enforced conditions were performed separately for each model. McNemar tests were used for paired per-note binary parse outcomes, including any final parse/schema failure across the three runs and final success across all three runs. Wilcoxon signed-rank tests were used for paired per-note median length ratio, run-to-run length-ratio variability, median numeric-token delta, median numeric-token preservation percentage, and median uncertainty-marker delta.

For the message-format sensitivity analysis, the primary endpoint was first-attempt invalid output, defined as parse failure or schema violation before retry. Secondary endpoints included persistent failure after retry, valid output after retry, code fences, leading text, and suspected token-cap truncation. Format effects were summarized as output-level counts and percentages and as paired note-level failure rates across the 10 repeated runs. Differences between the separate-role and embedded-user formats were assessed using Wilcoxon signed-rank tests for paired note-level rates and McNemar tests for note-level binary outcomes, including whether a note had at least one first-attempt invalid output or at least one persistent final failure. All inferential analyses were treated as exploratory screening comparisons or sensitivity checks rather than confirmatory hypothesis tests. The annotated R code for the primary analysis workflow is provided in Supplementary File S1, and the R code for the message-format sensitivity analysis is provided in Supplementary File S2.

### 2.7. Ethical Considerations

This study was approved by the Institutional Review Board of Dalin Tzu Chi Hospital, Buddhist Tzu Chi Medical Foundation (IRB No. B11402047-1). All admission notes used in this study were de-identified prior to analysis. Patient names, identification numbers, admission dates, and other personally identifiable information were removed from the source documents. Because this study involved only retrospective analysis of de-identified clinical documents using automated computational methods, with no direct patient contact or intervention, the requirement for individual informed consent was waived by the IRB. All model inference was performed on local hardware, and no patient data were transmitted to external servers or cloud-based services.

## 3. Results

### 3.1. Characteristics of the Evaluation Set

The evaluation set included 70 admission notes, with equal representation of internal medicine and surgical services. Descriptive text-level characteristics are shown in Table 3. Internal medicine notes were longer than surgical notes, with a median source-note length of 1233 characters after whitespace removal compared with 709 characters for surgical notes. Internal medicine notes also contained more numeric tokens, with a median of 23 compared with 11 in surgical notes. Uncertainty markers were infrequent overall, with a median count of 0 in both service groups. These characteristics indicate that the evaluation set included notes with variable length and quantitative clinical content, which is relevant to the interpretation of compression behavior and numeric-token loss in the model outputs.

### 3.2. Local Model Implementation and Structured-Output Validity

**Figure 2:**
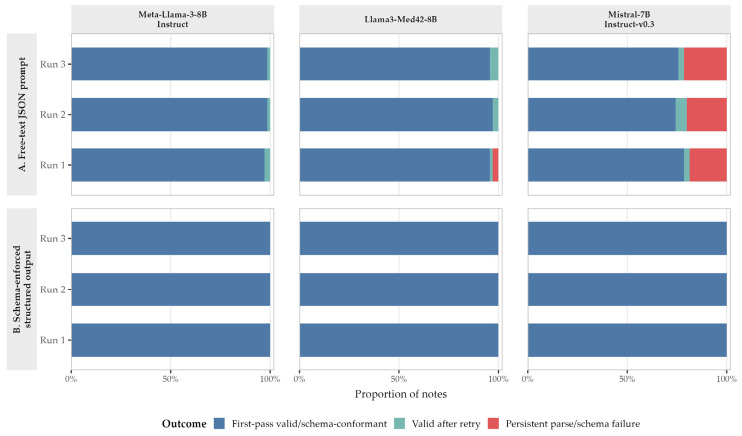
Structured-output stability across three independent inference runs under free-text JSON and schema-enforced structured-output conditions. Stacked bars show the proportion of 70 admission notes classified as first-pass valid JSON, valid after one retry, or persistent parse/schema failure for each model, run, and output-control condition. Persistent parse/schema failure indicates outputs that remained unparseable or schema-noncompliant after retry or verification.

All 1260 primary-study inferences were completed successfully using the local workflow described in Section 2.3 and Section 2.4. Structured-output validity differed substantially by output-control condition (Figure 2; Table 4). Under the free-text JSON prompt, the tested Mistral-7B-Instruct-v0.3/embedded-prompt configuration showed the weakest structured-output stability, producing first-pass valid and schema-compliant JSON for 52 to 55 of 70 notes per run (74.3–78.6%). An additional 2 to 4 notes per run (2.9–5.7%) became valid after one retry, whereas 13 to 15 notes per run (18.6–21.4%) remained unparseable or schema-noncompliant after retry. Llama3-Med42-8B produced first-pass valid JSON for 67 to 68 notes per run (95.7–97.1%), with 1 to 3 additional outputs per run becoming valid after retry and two persistent failures in Run 1 only. Meta-Llama-3-8B-Instruct produced first-pass valid JSON for 68 to 69 notes per run (97.1–98.6%); all remaining outputs became valid after retry and no persistent failures occurred. In contrast, under the schema-enforced structured-output condition, all three models produced 70 of 70 first-pass valid and schema-compliant outputs in all three runs.

Run-to-run stability further differentiated the output-control conditions. Under the free-text JSON prompt, Mistral-7B-Instruct-v0.3 had variable final status across runs for 10 of 70 notes (14.3%), at least one final parse/schema failure for 17 notes (24.3%), and consistent first-pass validity for 50 notes (71.4%). Ten Mistral notes failed after retry in all three runs. Llama3-Med42-8B had variable final status for 7 notes (10.0%), at least one final failure for 2 notes (2.9%), and consistent first-pass validity for 63 notes (90.0%). Meta-Llama-3-8B-Instruct had variable final status for 4 notes (5.7%), no final failures, and consistent first-pass validity for 66 notes (94.3%). Under schema enforcement, all three models achieved consistent first-pass validity for all 70 notes. Exploratory McNemar testing showed a significant paired reduction in Mistral final parse/schema failures under schema enforcement (*p* < 0.001), whereas Llama3-Med42-8B showed no statistically clear paired difference (*p* = 0.480) and Meta-Llama-3-8B-Instruct had no discordant final-failure pairs to test.

#### Message-Format Sensitivity Analysis

In the message-format sensitivity analysis, the embedded-user format produced 55 of 700 first-attempt invalid outputs (7.9%), compared with 12 of 700 outputs (1.7%) under the separate-role format. All invalid outputs were parse failures; no schema violations were observed. Persistent failure after one retry occurred in 35 of 700 outputs (5.0%) under the embedded-user format and 2 of 700 outputs (0.3%) under the separate-role format.

At the paired note level, the embedded-user format increased the first-attempt invalid-output rate by a mean of 6.1 percentage points (Wilcoxon signed-rank *p* = 0.035) and the persistent final-failure rate by 4.7 percentage points (*p* = 0.028). No suspected token-cap truncation was identified under either format. Because Mistral was not evaluated under both formats, this analysis could not exclude a model-by-format interaction.

### 3.3. Output-Token-Limit Assessment

The 1024-token output limit did not appear to constrain generation in the present evaluation set. No schema-valid output was classified as low-content, and all 630 schema-enforced outputs were structurally complete and first-pass schema-compliant. Considered together with the modest length of the source notes (maximum whitespace-stripped source-note length, 2364 characters; Table 3), these findings indicate that the 1024-token cap was adequate for this patient-note sample. This observation should not be extrapolated to substantially longer notes or to tasks requiring more extensive generation.

### 3.4. Documentation Behavior Among Structurally Valid Outputs

Documentation behavior was summarized for structurally valid outputs from both output-control conditions (Table 5). Under the free-text JSON condition, parseable schema-compliant outputs were available for 208 of 210 Llama3-Med42-8B outputs, 210 of 210 Meta-Llama-3-8B-Instruct outputs, and 168 of 210 Mistral-7B-Instruct-v0.3 outputs. Under schema enforcement, all 210 outputs from each model were parseable and schema-compliant. No output classified as schema-valid but low-content was detected in any model or condition.

#### 3.4.1. Instruction Compliance

Instruction compliance differed across both model and output-control condition. Under the free-text JSON prompt, among structurally valid outputs, Llama3-Med42-8B and Mistral-7B-Instruct-v0.3 did not produce code fences or leading text, whereas Meta-Llama-3-8B-Instruct wrapped 167 of 210 outputs (79.5%) in code fences and added leading text before the JSON object in 210 of 210 outputs (100%). These artifacts were recoverable by output cleaning, but they created a dependency on preprocessing before JSON parsing. Under schema enforcement, no code fences or leading text were detected in any model output, indicating that response-format control eliminated this specific formatting artifact in the local workflow.

#### 3.4.2. Verbosity Profile

Verbosity profiles remained model-specific after schema enforcement. Under the free-text JSON prompt, median length ratios were 0.82 for Llama3-Med42-8B, 1.00 for Meta-Llama-3-8B-Instruct, and 1.10 for Mistral-7B-Instruct-v0.3. Under schema enforcement, the corresponding medians were 0.81, 0.85, and 1.10. Thus, Llama3-Med42-8B remained compressive, Mistral-7B-Instruct-v0.3 remained length-neutral to mildly expansive among parseable outputs, and Meta-Llama-3-8B-Instruct shifted from a length-neutral profile to a more compressive profile. Exploratory paired Wilcoxon tests showed a significant reduction in per-note median length ratio for Meta-Llama-3-8B-Instruct under schema enforcement (*p* < 0.001), but not for Llama3-Med42-8B (*p* = 0.514) or Mistral-7B-Instruct-v0.3 (*p* = 0.874).

#### 3.4.3. Content Modification Signals

Content-modification signals also remained visible after schema enforcement. In the schema-enforced condition, median numeric-token deltas were −5 for Llama3-Med42-8B, −8 for Meta-Llama-3-8B-Instruct, and −2.5 for Mistral-7B-Instruct-v0.3. Median numeric-token preservation was 42.9%, 33.3%, and 70.0%, respectively. Compared with the free-text JSON prompt, schema enforcement was associated with lower numeric-token preservation for Meta-Llama-3-8B-Instruct (*p* < 0.001) and a more negative numeric-token delta (*p* < 0.001). Comparable paired differences were not detected for Llama3-Med42-8B or Mistral-7B-Instruct-v0.3. Uncertainty-marker deltas were centered at zero across all models and conditions. Because source uncertainty-marker counts were also near zero, this metric showed a strong floor effect and provided little discrimination between models or output-control conditions. The zero median deltas should therefore not be interpreted as evidence that the models consistently preserved uncertainty expression.

### 3.5. Summary of Model Profiles

The three models evaluated in this study exhibited distinct behavioral profiles with important implications for deployment suitability (Table 6). Under the free-text JSON prompt, the tested Mistral-7B-Instruct-v0.3/embedded-prompt configuration demonstrated low structural stability with persistent parse/schema failures and run-to-run variability, making it unsuitable for direct automated deployment without schema enforcement, retry mechanisms, or fallback infrastructure. Schema enforcement eliminated Mistral’s parse/schema failures in this task, but documentation-behavior signals still required review. Llama3-Med42-8B demonstrated high structural reliability and clean formatting under the free-text prompt, but its compression tendency and numeric-token loss suggest that revised notes may omit content from the source documentation. Meta-Llama-3-8B-Instruct achieved high structural reliability under both conditions, but under the free-text prompt, it systematically produced code fences and leading text; schema enforcement removed these artifacts while shifting the model toward a more compressive profile.

## 4. Discussion

### 4.1. Summary of Principal Findings

This study shows that pre-deployment suitability for locally deployed LLMs cannot be inferred from model size, domain fine-tuning, or single-run output quality alone. Using automated proxy metrics, we identified two distinct dimensions of deployment-relevant behavior: structured-output stability and documentation behavior. Under a free-text JSON prompt, the tested Mistral-7B-Instruct-v0.3/embedded-prompt configuration showed substantial structural instability, including persistent parse/schema failures and variable outcomes across identical runs. The message-format sensitivity analysis further showed that embedding system instructions in the user message increased first-attempt invalid outputs and persistent final failures in Meta-Llama-3-8B-Instruct, indicating that prompt message format itself can influence structured-output reliability. Schema enforcement eliminated these parsing failures for all three models in this task. However, structural success did not make the models equivalent: Llama3-Med42-8B remained compressive, Meta-Llama-3-8B-Instruct became more compressive under schema enforcement, and Mistral-7B-Instruct-v0.3 preserved numeric tokens more often among parseable outputs. These findings support a staged evaluation strategy in which automated structural screening and documentation-behavior profiling are performed before resource-intensive clinician review.

### 4.2. Structured-Output Stability in Context

The contrast between free-text prompting and schema-enforced output is central to the structured-output findings. Under the free-text JSON prompt, the relatively low first-pass parseability of the tested Mistral-7B-Instruct-v0.3/embedded-prompt configuration and the stochastic nature of its failures indicate that a prompt-only JSON instruction was not sufficient for reliable automated parsing in this local workflow. The same note could produce a valid output in one run and fail in another under identical conditions. However, this finding should not be attributed solely to Mistral model identity, because Mistral also differed from the two Llama models in message format.

The additional message-format sensitivity analysis addresses this concern directly. When Meta-Llama-3-8B-Instruct was tested with the same informational prompt content under two message formats, embedding system instructions within the user message increased first-attempt invalid outputs and persistent final failures compared with separate system and user roles. This supports the interpretation that message format can contribute to structural instability. At the same time, the sensitivity analysis does not fully isolate the cause of the original Mistral finding because Mistral could not be tested under both message configurations. The Mistral result should therefore be interpreted as a property of the tested Mistral/embedded-prompt configuration, with residual uncertainty about the independent contributions of model identity, message format, and their interaction.

The schema-enforced condition also informs the interpretation of the high consistency observed across the three runs. Under the free-text prompt, both Llama 3-based models were more stable than Mistral, but they still showed small numbers of variable first-pass or final statuses. Under schema enforcement, all three models produced consistent first-pass valid outputs for all 70 notes across all three runs. This does not guarantee that the same stability would hold at higher temperatures, with different prompt designs, with longer notes, or in other local inference engines, but it provides a practical baseline for operational planning. Structured-output stability also did not imply identical deployment suitability because documentation-behavior profiles differed across models and conditions.

### 4.3. Documentation Behavior Differences

The documentation behavior differences between Llama3-Med42-8B and Meta-Llama-3-8B-Instruct are notable because they emerged from models sharing the same Llama 3 8B base architecture and identical quantization, with fine-tuning strategy as the primary known difference between them. Llama3-Med42-8B, which was fine-tuned on medical-domain data, produced cleaner formatting under the free-text prompt and remained compressive across both output-control conditions. Meta-Llama-3-8B-Instruct, which was fine-tuned for general instruction following, produced more conversational formatting under the free-text prompt and shifted toward shorter outputs under schema enforcement. This pattern suggests that fine-tuning strategy and output-control method can jointly shape deployment behavior. The proxy metrics used in this study do not determine whether either behavior pattern is more clinically appropriate; they show where clinician review should focus.

Instruction compliance is operationally relevant because parseability after cleaning is not the same as pipeline readiness. Under the free-text prompt, Meta-Llama-3-8B-Instruct frequently wrapped JSON outputs in code fences and prepended explanatory text. Although these artifacts did not prevent parsing when output-cleaning logic was applied, they increased pipeline fragility. A production system expecting raw JSON would fail on many of these outputs without preprocessing. Schema enforcement eliminated these formatting artifacts in the present workflow, suggesting that response-format control can reduce middleware burden. Nevertheless, the same condition also changed documentation behavior for Meta-Llama-3-8B-Instruct, so structural gains should be checked against content-preservation signals.

The verbosity and numeric-token findings matter for deployment. Llama3-Med42-8B showed a consistent compression tendency, raising the possibility that revised notes may omit content from the original despite instructions not to alter clinical facts. Meta-Llama-3-8B-Instruct preserved note length more closely under the free-text prompt but became more compressive under schema enforcement and showed lower numeric-token preservation. Mistral-7B-Instruct-v0.3 was structurally unstable under the free-text prompt, yet under schema enforcement it showed a more length-neutral profile and higher median numeric-token preservation than the two Llama 3 models. None of these patterns is inherently preferable; the suitable model and output-control strategy depend on the intended use case and the institution’s tolerance for compression, formatting cleanup, and possible quantitative-content loss.

The present study did not include clinician adjudication of factual additions, omissions, or clinical inaccuracies; therefore, hallucination was not measured as a confirmed endpoint. The proxy metrics should instead be interpreted as signals of potential content-modification risk. For example, a large negative numeric-token delta may indicate that laboratory values, vital signs, medication doses, or dates were omitted during revision, whereas an extreme reduction in length ratio may suggest truncation or excessive summarization. Similarly, changes in uncertainty markers may indicate that the model altered the expressed level of diagnostic certainty. These signals do not prove hallucination or clinical error, but they identify outputs that warrant closer clinician review. In this sense, the proxy metrics function as screening indicators that can help triage model outputs for subsequent human evaluation rather than replace expert assessment of clinical correctness.

### 4.4. Proxy-Based Evaluation as a Pre-Deployment Screening Framework

Automated proxy metrics can provide a scalable first-stage screening tool for local LLM deployment by determining whether a candidate configuration meets basic operational requirements before clinician review. The proposed framework has two sequential functions. First, structural screening assesses whether outputs can be reliably captured, parsed, and transferred to downstream systems by examining JSON validity, schema adherence, run-to-run stability, instruction compliance, and dependence on output-cleaning middleware. Second, documentation-behavior profiling examines structurally valid outputs for patterns that may guide subsequent clinical review, including excessive compression, numeric-token change, quantitative-content loss, and changes in diagnostic-certainty language. This sequence distinguishes configurations that are technically unsuitable for automated processing from those that are structurally usable but still require focused clinician assessment.

The framework also emphasizes that output-control mechanisms should be evaluated as part of the complete deployment configuration. In the present study, schema enforcement changed structural reliability and formatting behavior while also affecting documentation-behavior signals. Candidate models should therefore be assessed together with the inference engine, chat-template handling, response-format controls, token limits, prompt structure, retry logic, and preprocessing rules. The message-format sensitivity analysis further showed that the placement of system instructions can affect structured-output reliability. For hospitals and research teams using local inference tools, such implementation choices can determine whether a configuration is suitable for an automated pipeline.

This screening approach complements human evaluation. Previous studies have reported that adapted LLMs can match or exceed expert-level performance on selected clinical text summarization tasks when completeness, correctness, conciseness, readability, and usefulness are assessed [18]. Such evaluations do not establish whether outputs can be generated consistently in a parseable and schema-compliant form within a local workflow. A model that produces clinically acceptable text may still require screening for JSON validity, instruction compliance, preprocessing requirements, and run-to-run instability. Conversely, passing proxy-based screening does not establish factual accuracy, completeness, safety, or usability, all of which still require clinician assessment.

Proxy-based screening also serves a different purpose from benchmark-based evaluation. Medical question-answering benchmarks, multitask evaluations, and clinical leaderboards assess model knowledge and reasoning, but they do not directly test structured-output stability within a specific documentation pipeline. The two forms of evidence are complementary: benchmarks can support candidate-model selection, whereas local proxy-based screening determines whether the selected configurations behave reliably under the intended deployment workflow.

### 4.5. Limitations

Several limitations should be considered when interpreting these findings. First, this study evaluated only three locally deployable models, all in the 7- to 8-billion-parameter range and implemented as 4 bit quantized GGUF models through LM Studio on consumer-grade hardware. The findings cannot be generalized to larger models, different architecture families, other local inference engines, or models accessed through cloud-based APIs with built-in structured-output enforcement. The selection was deliberate, representing models feasible for local deployment on consumer-grade hardware, but the behavioral patterns observed may not hold for models with greater capacity, different deployment environments, or different instruction-tuning procedures.

Second, all proxy metrics were computed automatically without human annotation. The metrics capture structural and behavioral properties of model outputs but do not assess clinical correctness, safety, completeness, or usability. A model that scores well on all proxy metrics may still produce clinically inappropriate content. The proxy-based framework identifies necessary but not sufficient conditions for deployment suitability.

Third, this study used one standardized prompt template, two output-control conditions, and a fixed set of inference parameters, including temperature = 0.2 and a maximum generated-output limit of 1024 tokens. The final prompt was developed through author consensus and refined for clarity and brevity with AI assistance, but no broader prompt-ablation or prompt-comparison experiment was performed. This design supported comparability across models and allowed comparison between free-text JSON prompting and schema-enforced structured output, but it did not evaluate whether alternative prompts, few-shot examples, shorter schema descriptions, model-specific prompt adaptations, different schema-enforcement configurations, or different output-token limits could further improve structured-output reliability or documentation behavior. Although the 1024-token output limit did not appear to be a binding constraint in this note sample, longer admission notes, discharge summaries, or tasks requiring more extensive rewriting may require a larger output budget and explicit monitoring for length-based stopping or truncation. Therefore, the findings should be interpreted as model behavior under one standardized pre-deployment prompt, two output-control conditions, and one fixed inference configuration, rather than as the best achievable performance of each model.

Fourth, the primary repeated-run design used only three independent generations per note, model, and output-control condition. This was sufficient to detect overt run-to-run instability but was not sufficient to estimate rare failure probabilities with precision, especially at the low temperature setting of 0.2. Low-temperature generation reduces sampling variability but does not make output deterministic. Therefore, absence of observed variation across three runs should not be interpreted as evidence of deterministic stability. The 10-run message-format sensitivity analysis partly addresses this issue for one model and two message formats, but larger repeated-run evaluations would be needed to estimate uncommon instability rates more precisely.

Fifth, the evaluation set included only 70 English-language admission notes drawn from a single institution in Taiwan, and all notes were originally written by nurse practitioners. The notes were limited to internal medicine and surgical services, and the findings may not generalize to other clinical specialties, documentation styles, healthcare systems, languages, or professional author groups. The generalizability of the observed behavioral profiles to other clinical documentation contexts remains to be established.

Sixth, model identity and message format were not completely separable in the primary comparison. Although the Meta-Llama sensitivity analysis showed that embedded system instructions can increase structural instability, Mistral could not be tested under both formats; model-by-format interaction therefore remains possible.

Seventh, the proxy metrics used in this study have not been validated against downstream clinical outcomes or clinician judgments of documentation quality. Whether instruction compliance rate, length ratio, or numeric-token delta predict clinically meaningful differences in documentation quality is an empirical question that this study was not designed to answer. Future research should examine the correlation between proxy metrics and human quality assessments to establish predictive validity.

Eighth, the uncertainty-marker metric had limited discriminative value because explicit uncertainty terms were uncommon and the dictionary-based method could not capture semantic changes in claim strength.

### 4.6. Implications for Practice and Future Research

For healthcare institutions considering local deployment of LLMs for clinical documentation, model selection should not rely solely on parameter size, benchmark performance, or domain-specific fine-tuning claims. Candidate models should first be tested under the intended local workflow, including the same inference engine, quantization format, prompt structure, output schema, and hardware configuration planned for deployment. In this study, all three models were similar in size and were evaluated under the same local environment, yet their structured-output stability and documentation behavior differed substantially.

A practical deployment pathway would begin with automated screening for parse failures, schema noncompliance, unstable per-note outcomes, instruction noncompliance, excessive compression, numeric-token loss, and changes in uncertainty language. Models with frequent parse failures or unstable outputs may require schema enforcement, retry logic, fallback mechanisms, or grammar-constrained decoding before further evaluation. Models with systematic formatting artifacts, such as code fences or leading text, may remain usable if reliable output-cleaning middleware is implemented, although response-format control may reduce that need. Models with compression tendencies or numeric-token loss should undergo focused clinician review to determine whether omitted content is redundant or clinically important.

These findings also support incorporating repeated-run testing into pre-deployment evaluation protocols. The variable outcomes observed for Mistral-7B-Instruct-v0.3 under the free-text JSON prompt show that single-run evaluation may underestimate operational instability. Repeating inference on the same inputs requires additional compute time but can reveal stochastic behavior that would otherwise appear unpredictably in production. Repeated-run testing is also useful after schema enforcement because it confirms whether the apparent structural reliability persists across repeated executions and whether documentation-behavior signals remain stable.

Beyond admission-note editing, the same staged evaluation logic may apply to other clinical documentation tasks that require structured outputs, including discharge summary drafting, referral-letter revision, problem-list generation, structured data extraction, and form-filling workflows. However, task-specific proxy metrics would need to be adapted to each use case. For example, numeric-token change and uncertainty-marker change are particularly relevant to clinical documentation, whereas other domains may require different content-preservation indicators.

Future research should extend this framework by evaluating larger models, additional architecture families, and alternative local inference engines. Further studies should also examine prompt sensitivity, decoding settings, output-token limits, and grammar-constrained generation. Proxy metrics should be validated against blinded clinician assessments to determine whether instruction compliance, compression tendency, numeric-token loss, and uncertainty-marker changes predict clinically meaningful differences in documentation quality.

## 5. Conclusions

This proxy-based pre-deployment evaluation showed that structured-output reliability depends on the complete model, prompt message, and output-control configuration. Prompt-only JSON generation was insufficient for the tested Mistral-7B-Instruct-v0.3/embedded-prompt configuration, and the message-format sensitivity analysis showed that embedding system instructions within the user message can increase structural instability when model identity is held constant. Schema enforcement eliminated parse/schema failures across all tested models and runs, although model-specific differences in compression and numeric-token preservation remained. Automated structural screening and documentation-behavior profiling should therefore precede clinician assessment of clinical correctness, safety, and usability.

## Figures and Tables

**Table 1:** Summary of related work and positioning of the present study.

Area of Related Work	Representative References	Dataset/Task and Models/Systems	Main Focus or Findings	Remaining Gap Addressed in the Present Study
Clinical documentation and summarization	[4,5,6,7]	Clinical notes, documentation workflows, and summarization tasks using AI documentation systems or adapted LLMs	Prior studies mainly evaluated clinical quality, readability, completeness, summarization quality, or clinician-rated usefulness	These studies do not directly test whether outputs are structurally valid and pipeline-ready before clinician review
Medical LLM benchmarks and healthcare LLM systems	[1,2,3]	Medical question-answering and multitask reasoning benchmarks using general or medical-domain LLMs	Benchmarks assess medical knowledge, reasoning, and question-answering performance	Benchmark performance may not predict JSON validity, schema adherence, or run-to-run stability in a local documentation pipeline
Local and privacy-preserving LLM deployment	[8,9]	Local clinical text-generation or documentation tasks using locally deployed or fine-tuned open-weight LLMs	Local deployment can reduce external data transmission and support privacy-preserving clinical use	Local deployment introduces hardware, quantization, and chat-template constraints that require direct operational testing
Structured-output generation	[10,11,12,13,14]	Structured extraction, JSON/XML/YAML generation, and constrained-output tasks using small LLMs or constrained-generation methods	Prior work shows that parseability, schema adherence, and format restrictions can affect model behavior and output usability	Limited evidence exists for structured admission-note editing using locally deployed 7–8B models
Present study	—	70 de-identified admission notes, including 35 internal medicine and 35 surgical notes, evaluated using Llama3-Med42-8B, Meta-Llama-3-8B-Instruct, and Mistral-7B-Instruct-v0.3 under free-text JSON and schema-enforced structured-output conditions	Evaluates structured-output stability, run-to-run consistency, instruction compliance, verbosity, and content-modification signals under two output-control conditions	Provides a proxy-based pre-deployment framework for identifying unstable model-prompt configurations and compare structural mitigation strategies before resource-intensive clinician evaluation

**Table 2:** Model specifications.

Model	Base Architecture	Parameters	Fine-Tuning Domain	Source Repository	Quantization (File Size)
Llama3-Med42-8B	Llama 3	8B	Medical (clinical)	QuantFactory/Llama3-Med42-8B-GGUF	Q4_K_S (4.37 GB)
Meta-Llama-3-8B-Instruct	Llama 3	8B	General instruction	MaziyarPanahi/Meta-Llama-3-8B-Instruct-GGUF	Q4_K_S (4.37 GB)
Mistral-7B-Instruct-v0.3	Mistral 7B	7B	General instruction	Mistralai/Mistral-7B-Instruct-v0.3-GGUF	Q4_K_S (4.07 GB)

GGUF: GPT-Generated Unified Format.

**Table 3:** Descriptive characteristics of the admission-note evaluation set.

Characteristic	Overall (n = 70)	Internal Medicine (n = 35)	Surgery (n = 35)
Source-note length, median (IQR)	943.5 (693.5–1260.5)	1233 (931–1432)	709 (629.5–969.5)
Source-note length, range	437–2364	576–2364	437–1935
Numeric-token count, median (IQR)	12 (8–28)	23 (10.5–31)	11 (5.5–18)
Numeric-token count, range	2–105	4–105	2–36
Uncertainty-marker count, median (IQR)	0 (0–0)	0 (0–1)	0 (0–0)
Uncertainty-marker count, range	0–4	0–2	0–4

Source-note length was calculated after removing all whitespace. Numeric tokens and uncertainty markers were extracted and counted as defined in Section 2.5.2. IQR = interquartile range.

**Table 4:** Structured-output stability and JSON parsing outcomes across three independent inference runs by output-control condition (n = 70 notes per run).

Condition	Model	Run 1	Run 2	Run 3
Free-text JSON prompt	Llama3-Med42-8B	67/1/2	68/2/0	67/3/0
Free-text JSON prompt	Meta-Llama-3-8B-Instruct	68/2/0	69/1/0	69/1/0
Free-text JSON prompt	Mistral-7B-Instruct-v0.3	55/2/13	52/4/14	53/2/15
Schema-enforced structured output	Llama3-Med42-8B	70/0/0	70/0/0	70/0/0
Schema-enforced structured output	Meta-Llama-3-8B-Instruct	70/0/0	70/0/0	70/0/0
Schema-enforced structured output	Mistral-7B-Instruct-v0.3	70/0/0	70/0/0	70/0/0

Each cell reports first-pass valid JSON/valid after retry/persistent parse/schema failure. The Mistral free-text results reflect the tested Mistral-7B-Instruct-v0.3/embedded-prompt configuration.

**Table 5:** Documentation behavior proxy metrics by output-control condition and model, calculated from parseable schema-compliant outputs across all three runs.

Condition	Model	Parseable Outputs	Length Ratio, Median (IQR)	Numeric Delta, Median (IQR)	Numeric Preservation, Median (IQR), %	Uncertainty Delta, Median (IQR)
Free-text JSON prompt	Llama3-Med42-8B	208/210 (99%)	0.82 (0.65, 1)	−5 (−14, −1)	50 (25, 72.6)	0 (0, 0)
Free-text JSON prompt	Meta-Llama-3-8B-Instruct	210/210 (100%)	1 (0.78, 1.12)	−6 (−13, −2)	50 (30.6, 71.4)	0 (0, 0)
Free-text JSON prompt	Mistral-7B-Instruct-v0.3	168/210 (80%)	1.1 (0.99, 1.21)	−2 (−7, −1)	71.4 (59.8, 83.9)	0 (0, 0)
Schema-enforced structured output	Llama3-Med42-8B	210/210 (100%)	0.81 (0.62, 1.01)	−5 (−15, −1)	42.9 (27, 72.2)	0 (0, 0)
Schema-enforced structured output	Meta-Llama-3-8B-Instruct	210/210 (100%)	0.85 (0.61, 1.03)	−8 (−19.8, −2)	33.3 (15.5, 62.5)	0 (0, 0)
Schema-enforced structured output	Mistral-7B-Instruct-v0.3	210/210 (100%)	1.1 (0.99, 1.21)	−2.5 (−7, −1)	70 (57.2, 80)	0 (0, 0)

Metrics were calculated only for parseable, schema-compliant outputs. Length ratio was based on whitespace-stripped character counts. Numeric-token delta, numeric-token preservation, and uncertainty-marker delta were calculated as defined in Section 2.5.2. IQR = interquartile range.

**Table 6:** Screening-based interpretation of deployment-relevant model behavior by output-control condition.

Dimension	Mistral-7B-Instruct-v0.3/Embedded-Prompt Configuration	Llama3-Med42-8B	Meta-Llama-3-8B-Instruct
Free-text structural stability	Low: persistent failures in all runs; 17/70 notes had at least one final failure	High: two final failures in Run 1 only; 68/70 notes final-successful in all runs	High: no final failures; code fences/leading text common
Schema-enforced structural stability	High: 70/70 first-pass valid in all runs	High: 70/70 first-pass valid in all runs	High: 70/70 first-pass valid in all runs
Main residual issue	Needs schema enforcement or alternative message-format handling for reliable parsing; content review still needed	Compression tendency and numeric-token loss	Free-text formatting artifacts; lower length ratio and numeric preservation under schema enforcement
Pipeline requirement	Schema enforcement, retry/fallback if free-text prompting is used	Clinical content verification, especially for numeric details	Schema enforcement or output-cleaning middleware; clinical content verification
Overall screening interpretation	Unsuitable for direct automated pipeline use under the tested free-text embedded-prompt configuration; reconsider after schema enforcement or alternative message-format handling	Suitable for clinician review after content-preservation checks	Suitable for clinician review, preferably with schema enforcement and content-preservation checks

## Data Availability

The de-identified clinical-note evaluation set is not publicly available because it was derived from institutional clinical documentation and remains subject to institutional data-governance. The derived summary data and analysis code are available from the corresponding author upon reasonable request, subject to institutional approval.

## Supplementary Materials

The following supporting information can be downloaded at: https://www.mdpi.com/article/10.3390/healthcare14142150/s1. Supplementary File S1: Prompt structures and annotated R code for the primary analysis workflow. Supplementary File S2: R code for message-format sensitivity analysis. Supplementary Table S1: Proxy metric definitions and extraction rules.

## Abbreviations

The following abbreviations are used in this manuscript:

AI	Artificial intelligence
API	Application programming interface
DDR RAM	Double data rate random access memory
GGUF	GPT-Generated Unified Format
GPU	Graphics processing unit
IQR	Interquartile range
JSON	JavaScript Object Notation
LLM	Large language model
VRAM	Video random access memory
XML	Extensible Markup Language
YAML	YAML Ain’t Markup Language (originally “Yet Another Markup Language”)

## References

[1] Lin C., Kuo C.F. (2025). Roles and potential of large language models in healthcare: A comprehensive review. Biomed. J..

[2] Google Med-PaLM. https://sites.research.google/med-palm/.

[3] Microsoft Microsoft Dragon Copilot. https://www.microsoft.com/en-us/health-solutions/clinical-workflow/dragon-copilot.

[4] Bracken A., Reilly C., Feeley A., Sheehan E., Merghani K., Feeley I. (2025). Artificial intelligence (AI)-powered documentation systems in healthcare: A systematic review. J. Med. Syst..

[5] Balloch J., Sridharan S., Oldham G., Wray J., Gough P., Robinson R., Sebire N.J., Khalil S., Asgari E., Tan C. (2024). Use of an ambient artificial intelligence tool to improve quality of clinical documentation. Future Healthc. J..

[6] Lee C., Britto S., Diwan K. (2024). Evaluating the impact of artificial intelligence (AI) on clinical documentation efficiency and accuracy across clinical settings: A scoping review. Cureus.

[7] Stetson P.D., Bakken S., Wrenn J.O., Siegler E.L. (2012). Assessing electronic note quality using the Physician Documentation Quality Instrument (PDQI-9). Appl. Clin. Inform..

[8] Dennstädt F., Hastings J., Putora P.M., Schmerder M., Cihoric N. (2025). Implementing large language models in healthcare while balancing control, collaboration, costs and security. npj Digit. Med..

[9] Hou Y., Bert C., Gomaa A., Lahmer G., Höfler D., Weissmann T., Voigt R., Schubert P., Schmitter C., Depardon A. (2025). Fine-tuning a local LLaMA-3 large language model for automated privacy-preserving physician letter generation in radiation oncology. Front. Artif. Intell..

[10] Neveditsin N., Lingras P., Mago V. (2025). Evaluating structured output robustness of small language models for open attribute-value extraction from clinical notes. arXiv.

[11] Tam Z.R., Wu C.K., Tsai Y.L., Lin C.Y., Lee H.Y., Chen Y.N. (2024). Let me speak freely? A study on the impact of format restrictions on performance of large language models. arXiv.

[12] Galeone C., Park M., Ettorre G., Ligorio D. (2026). When correct isn’t usable: Improving structured output reliability in small language models. arXiv.

[13] Willard B.T., Louf R. (2023). Efficient guided generation for large language models. arXiv.

[14] Geng S., Cooper H., Moskal M., Jenkins S., Berman J., Ranchin N., West R., Horvitz E., Nori H. (2025). Generating structured outputs from language models: Benchmark and studies. arXiv.

[15] QuantFactory Llama3-Med42-8B-GGUF. https://huggingface.co/QuantFactory/Llama3-Med42-8B-GGUF.

[16] MaziyarPanahi Meta-Llama-3-8B-Instruct-GGUF. https://huggingface.co/MaziyarPanahi/Meta-Llama-3-8B-Instruct-GGUF.

[17] Mistral AI Mistral-7B-Instruct-v0.3. https://huggingface.co/mistralai/Mistral-7B-Instruct-v0.3.

[18] Van Veen D., Van Uden C., Blankemeier L., Delbrouck J.B., Aali A., Bluethgen C., Pareek A., Polacin M., Reis E.P., Seehofnerová A. (2024). Adapted large language models can outperform medical experts in clinical text summarization. Nat. Med..

