# Supplementary material for: Schema Enforcement and Structured-Output Stability in Locally Deployed LLMs for Clinical Admission-Note Editing: A Proxy-Based Pre-Deployment Evaluation
Source: Healthcare (Basel). 2026 Jul 16;14(14):2150. doi: 10.3390/healthcare14142150 (PMC13410419; doi:10.3390/healthcare14142150)
Supplement: Supplementary file 1 [file healthcare-14-02150-s001.zip › Supplementary Table S1.pdf]

Supplementary Table S1. Proxy metric definitions and extraction rules

Each metric was computed automatically in R from the stored model outputs and the source notes, without human annotation. Regular expressions are shown in R syntax (case-insensitive where noted).

<table>
<tr><th>Metric</th><th>Definition</th><th>Exact rule or regular expression</th></tr>
<tr><td>Output status (stability)</td><td>First-pass response parsed and schema-checked. ok = parseable and schema-conformant on first attempt; ok_after_retry = conformant only after one repeat inference; fail = not conformant after retry.</td><td>Extract first balanced {...} (brace counting, quotes ignored); repair triple-quoted revised_note; parse with jsonlite::fromJSON; conformant = exactly the 4 keys, no missing or extra, revised_note a string, other 3 arrays of strings.</td></tr>
<tr><td>Instruction compliance</td><td>Whether the raw output carried formatting that violated the return-only-JSON instruction (code fences or leading text before the JSON object).</td><td>has_code_fence: str_detect(text, fixed("```")). has_leading_text: any non-whitespace text before the first {.</td></tr>
<tr><td>Verbosity profile (length ratio)</td><td>Whitespace-stripped character count of revised_note divided by that of the source note. <1 compression, ~1 neutral, >1 expansion.</td><td>nchar(gsub("\\s+","",revised)) / nchar(gsub("\\s+","",source)) (source count floored at 1).</td></tr>
<tr><td>Numeric-token delta</td><td>Count of numeric tokens in revised note minus count in source note. Negative = fewer numbers after revision.</td><td>Tokens via \b\d+(\.\d+)?\b. Digits fused to letters (T2DM, HbA1c) not matched; digits split by a non-word character (the 19 in COVID-19, hyphenated dates/ranges) matched as separate tokens.</td></tr>
<tr><td>Numeric-token preservation</td><td>Percentage of source numeric tokens that reappear in the revised note, matched by value with multiplicity and independent of position.</td><td>100 × |multiset(source) ∩ multiset(revised)| / |multiset(source)|, using the same \b\d+(\.\d+)?\b tokens; NA when the source has no numeric tokens.</td></tr>
<tr><td>Uncertainty-marker delta</td><td>Count of uncertainty markers in revised note minus count in source note.</td><td>Matches per note = \b(possible|possibly|suspect(?:ed)?|r/o|rule\s*out|likely|consider)\b (case-insensitive) plus one count per ? character.</td></tr>
<tr><td>Schema-valid but low-content (flag)</td><td>Schema-valid output carrying no usable revised text: revised_note empty or a placeholder, or array fields empty or all placeholders. Reported separately.</td><td>Placeholder set (case-insensitive): na, none, null, not applicable, not available, unknown, no information, no change(s), not specified, empty, placeholder.</td></tr>
</table>

Uncertainty-marker matching is whole-word; inflections outside the listed alternations (for example “ruled out,” “ruling out,” “suspicious,” “suspicion”) are not matched. All extraction was applied identically to source and revised notes.
